# Perspective: Towards single shot time-resolved microscopy using short wavelength table-top light sources

**DOI:** 10.1063/1.5082686

**Published:** 2019-02-05

**Authors:** T. Helk, M. Zürch, C. Spielmann

**Affiliations:** 1 Institute of Optics and Quantumelectronics, Abbe Center of Photonics, Friedrich-Schiller-University, 07743 Jena, Germany; 2 Helmholtz Institute Jena, 07743 Jena, Germany; 3 Fritz Haber Institute in the Max Planck Society, 14195 Berlin, Germany

## Abstract

Time-resolved imaging allows revealing the interaction mechanisms in the microcosm of both inorganic and biological objects. While X-ray microscopy has proven its advantages for resolving objects beyond what can be achieved using optical microscopes, dynamic studies using full-field imaging at the nanometer scale are still in their infancy. In this perspective, we present the current state of the art techniques for full-field imaging in the extreme-ultraviolet- and soft X-ray-regime which are suitable for single exposure applications as they are paramount for studying dynamics in nanoscale systems. We evaluate the performance of currently available table-top sources, with special emphasis on applications, photon flux, and coherence. Examples for applications of single shot imaging in physics, biology, and industrial applications are discussed.

## I. INTRODUCTION

Exploring and understanding the functionality and dynamics in molecules, thin films, biological cells, and clusters are crucial for advancing the current technology for medical, physical, or chemical applications. The smallest achievable resolution in full-field imaging Δ*r* is limited and can be estimated with the Abbe criterion, $\Delta r=\frac{\lambda }{2\cdot \text{NA}}$, depending on the incident wavelength *λ* and the numerical aperture NA [NA = n × sin(*α*), with n being the refractive index of the medium between the object and the objective and *α* the opening angle of the collected light cone] of the optical system. In a conventional visible light microscope, a resolution down to 0.2 *μ*m can be achieved for the highest possible NA of about 1. In the last few years, we have witnessed a tremendous progress in this area, resulting in optical imaging with a higher resolution under restricted conditions.

With Stochastic Optical Reconstruction Microscopy (STORM),^1^ Stimulated Emission Depletion (STED),^2,3^ and Nearfield Scanning Optical Microscopy (NSOM),^4,5^ it is now possible to reach a resolution on the order of 20 nm.^4,5^ NSOM, STED, and STORM are only a few examples of novel microscopy methods combining advanced numerical methods for image processing with raster scanning techniques and/or spatial modulation of the incident field to overcome the diffraction limit. However, for this improvement, certain constraints are required such as marking the object with specially designed fluorophores, inserting a sufficiently small aperture, reducing the overall light transmission, or controlling the motion of apertures or objects with nanometer precision signifying the difference in full-field imaging. Inspecting the Abbe criterion reveals a more straightforward approach to resolve nanoscopic objects, namely, shortening the wavelength of the illuminating radiation. Using radiation in the sub-keV regime, the extreme ultraviolet (XUV) and soft X-ray radiation (SXR), between 1–10 nm (SXR) and 10–100 nm (XUV), respectively, can improve the resolution by several orders of magnitude compared to optical microscopy. Specifically, for the investigation of a biological specimen, radiation in the so-called *water window*^6^ between 2.32 nm and 4.37 nm corresponding to the K-absorption edges of oxygen and carbon is very well suited to enable high contrast images, which are the key for understanding the inner mechanisms of cells. In addition to biological relevance, the investigation of the laser-solid interaction using time-resolved imaging is another emerging application of high-resolution real-time microscopy.

The wide variety of powerful imaging techniques developed for the visible spectral range cannot easily be transferred into the XUV/SXR-range due to the strong absorption or losses of the available optical components. Reflective optics in grazing incidence, multilayer mirrors, and Fresnel-zone-plates is the choice for efficient use of the incoming radiation. However, the rather low NA and/or inherent aberrations of all these optical components limit the achievable resolution. A more conventional X-ray transmission microscope with zone-plates as imaging optics can resolve structures as small as 15 nm^7^ for an illumination wavelength of 1.52 nm and an effective NA of 0.05. A powerful way to overcome the restrictions by imaging optics is using lensless imaging approaches such as Fourier-transform-holography^8^ (FTH) and coherent diffractive imaging (CDI)^9^ and its extension ptychography.^10,11^

Combining nanometer spatial resolution imaging with time-resolved methods appears especially appealing for monitoring objects inside the materials and following their motion and their modifications.^12^ Tracking the motion of, e.g., molecular clusters inside cells and studying their subsequent interlinking mechanisms provide information about the fundamental processes of life. All of these processes happen on their typical time scale: inorganic objects and organic species move in liquids on a time scale of seconds to microseconds, structural changes such as melting or solidification of solids occur on the nanosecond to picosecond timescales, the dissociation of molecules is linked to the femtosecond-range, and finally to follow motion of electrons, attosecond resolution is required. Time-resolved measurements of modifications or motions in inorganic or biological system materials are usually triggered by an intense ultrashort laser pulse, and the subsequent evolution of the system is observed by taking a series of snapshots with variable delay. In this approach, the temporal resolution is defined by the exposure time, i.e., the duration of the illuminating light pulse. Ideally, each pulse of the illuminating radiation has sufficient flux for imaging the specimen in a single shot. Realizing a light source delivering radiation in ultrashort pulses and high energy photons will be very beneficial for gaining new insights into these processes of the microcosm.

In this perspective, we review the current efforts towards single shot XUV/SXR-microscopy. We will also discuss the recent advances in the development of table-top ultrafast short wavelength sources, enabling new microscopic applications in science and technology.

## II. IMAGING METHODS IN XUV/SXR

The most common XUV/SXR-microscopy approaches are either more conventional microscopy with a zone-plate as imaging optic or lensless methods for retrieving the information about the object from the diffracted light. To be mentioned for the sake of completeness, more recently, another method called ghost imaging^13,14^ has been reported in the literature, enabling imaging in the X-ray and optical regime. Ghost imaging is not a single shot method since it requires recording many individual strongly undersampled images as basis for retrieving the object by calculating the correlation between the images. Specifically, for short wavelength radiation, ghost imaging is attractive because it allows low-dose shadowgraphy. Table-top short wavelength imaging transmission microscopy using zone plates is a versatile and robust approach which is widely used, whether the source is coherent or incoherent. The commonly used X-ray source is a laser produced plasma (LPP)^15–18^ that emits either line or continuous radiation with a low degree of spatial coherence. In any case, the radiation must be spectrally filtered to ensure monochromatic illumination. Monochromacy is essential because zone-plates have a strong chromatic aberration limiting, otherwise, the achievable resolution. The radiation is focused onto the sample, and the outgoing field from the object is collected by a zone-plate and imaged onto a 2D-detector,^7,19,20^ such as a charged coupled device. As, e.g., LPP based on the interaction with a liquid nitrogen jet is a brilliant source in the few nanometer wavelength range,^19,21^ this imaging technique is especially suitable in the water window, providing a large amplitude contrast for biological samples in their natural environment. The resulting image shows only the wavelength and thickness dependent absorption of the object. The inherent loss of 3D is outweighed by an easily adjustable magnification (changing the distances between the object, the zone-plate, and the detector) and a comparatively low photon flux on the sample for achieving a high-quality image compared to lensless methods such as holography.^22^ All the drawbacks linked to zone-plates can be avoided by employing lensless imaging techniques. FTH and CDI have been successfully implemented in the XUV/SXR-range using light sources in a wide parameter range including large-scale facilities [synchrotrons and Free-Electron-Lasers (FEL)] and more recently compact table-top approaches [high harmonic generation^23^ (HHG) or soft X-ray-lasers^24^], fitting a university size laboratory.

The main challenge of these lensless imaging techniques is the reconstruction of the object from the diffracted light directly recorded with a 2D-detector. As such, detectors can only record the intensity distribution, and the so-called phase problem must be solved to obtain the object. In FTH, the wave diffracted by the sample is superimposed with a known reference wave, usually generated by a pinhole prepared in the same plan as the object. The interference pattern between these two waves is recorded^25^ and termed the hologram. Finally, a numerical 2D-Fourier-transform of the measured holograms recovers the object. However, FTH requires a matching of the distance between the origin of the reference wave (pinhole) and the object, hence requiring *a priori* knowledge of the object dimensions. A more versatile implementation is digital-inline-holography, where a curved wavefront illuminates the object and acts as a reference wave at the same time. A major limitation of all holographic techniques is that the size of the pinhole determines the achievable resolution of the image. However, reducing the pinhole diameter results in significantly reduced transmission which must be compensated with a longer exposure time. Imaging with CDI omits the need for generating a reference wave by recording only the light diffracted by the object and subsequently solving the phase problem numerically. For all algorithms, the best match is searched between the measured intensity distribution and the calculated one, assuming different phase profiles. The phase problem can be solved for an isolated object by oversampling the diffraction pattern on the detector, satisfying the Nyquist-Shannon-criterion.^26^ The most widely used phase retrieval algorithm starts initially by assuming plane wave illumination and a random phase distribution for obtaining the first guess of the intensity distribution on the detector. To constrain for an isolated object, the so-called support is enforced in the object space during the iterative phase retrieval. The autocorrelation of the intensity pattern can be used to obtain a first approximation of the shape of the support (i.e., estimation of the size and shape of the object) for the retrieval. The phase is iteratively retrieved by varying the phase after Fourier transforming the complex fields between the Fourier and object domain iteratively, where the measured amplitudes in the Fourier domain and the finite support in the object domain are enforced.^27^ Besides several improvements and the basic Gerchberg-Saxton type algorithm,^28^ the dynamic adaption of the support called shrink-wrap^29^ leads to better recovery of objects from experimental data. The key benefit of CDI is that the resolution depends only on the wavelength of the light source and the NA of the setup, i.e., the relationship between the detector size and the object-to-detector distance. Furthermore, CDI is one of the most photon efficient techniques, given that lossy optical components are omitted. Despite the simplicity of the setup, the experimental setup must meet several boundary conditions. The recorded pattern must fulfil the oversampling condition, as stated by Sayre,^30^ at least by a factor of two in each dimension, i.e., the sample must be a factor of two smaller than the illuminated area. CDI further requires illumination with spatially and temporally coherent radiation. Like the condition provided by Sayre, Spence *et al.* provided estimates for the minimum coherence lengths.^31^ The spatial coherence length must be at least twice the maximum object feature size, and the temporal/longitudinal coherence length must be longer as the maximum pathway difference between two distant points of the object. So, the coherence of the source is a crucial criterion for implementing lensless techniques in XUV/SXR-imaging. Specifically, the temporal coherence must suffice certain conditions, which hamper the general temporal resolution. For the highest spatial resolution, the longitudinal coherence *l_c_* should be, if possible, implying a nearly monochromatic light source. On the other hand, the duration of ultrashort light pulses should be as low as possible to ensure the highest temporal resolution. However, for ultrashort light pulses, the duration *τ* is $\propto \frac{1}{\Delta \nu }$, which is the spectral width. Hence, for experiments, a trade-off must be made between the achievable spatial and temporal resolution. The requirement for isolated objects in CDI can be relaxed by using ptychography.^32^ Ptychography relies on the redundancy of information of many diffractograms gathered from overlapping illumination spots and the knowledge of relative positioning of the focal spot on the sample, and one can recover arbitrarily the extended objects. The large redundancy of diffraction data additionally allows us to recover the illumination function. This knowledge in general allows higher fidelity in imaging^10,33^—in contrast to CDI, where normally simply a plane wave is assumed as the illumination function. On the other hand, ptychography poses some additional requirements for the source such as a good pointing stability to ensure well-defined relative positioning of the subsequent illumination spots. This specific requirement limits the applicability of X-ray ptychography in current FEL sources. All mentioned techniques can be combined with a rotating sample, enabling, instead of 2D-, 3D-image reconstruction or tomography. Recent examples for 3D-CDI,^34–36^ 3D-ptychography,^11,37^ and 3D-zone-plate imaging^21,38^ demonstrate the exquisite capabilities of these techniques.

## III. LIGHT SOURCES WITH POTENTIAL FOR SINGLE SHOT WATER WINDOW IMAGING

**FIG. 1.**
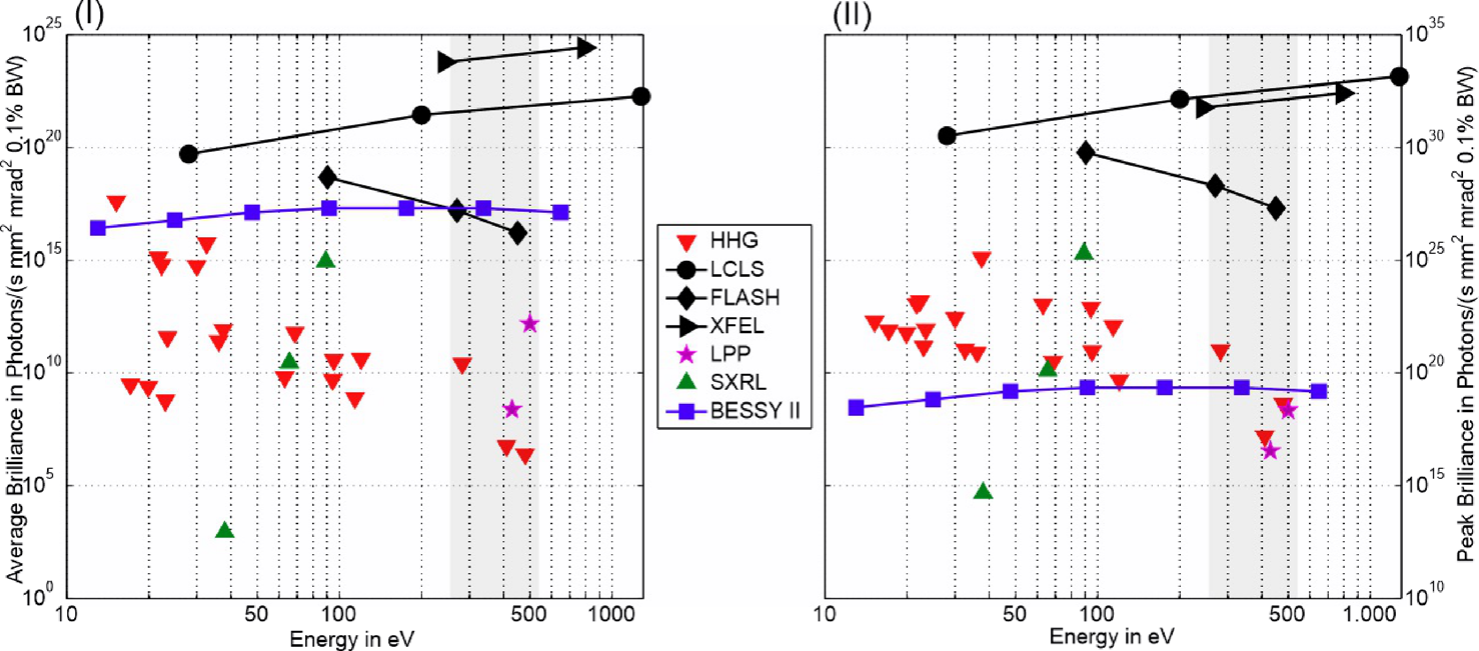
Comparison of the (I) average brilliance and (II) peak brilliance of different XUV/SXR-sources vs. photon energies. The grey highlighted area indicates the water window. The data are taken from recently published experimental results for table-top sources (HHG,^52–65^ SXRL,^24,66,67^ and LPP^16,48^) and large-scale facilities (synchrotron BESSY II^56^ and X-FELs such as LCLS, European XFEL, and FLASH^42,68,69^).

High-quality imaging with the aspired resolution requires a minimum number of photons on the detector. For many applications based on less brilliant table-top light sources, this requirement can be easily met by increasing the exposure time accordingly. However, for time-resolved imaging measurements in a pump-probe setup, the necessary number of photons must be provided in a single shot. Also, for biological samples, the maximum exposure time is limited because the high energy photons can cause radiation damage inside cells followed by disintegration.^39^ Thus, using several pulses or longer integration time would inevitably lead to modification of the observed structures and a subsequent blurring of the reconstructed image. Solem^22^ and Schneider^40^ estimated for different types of cells and imaging techniques the required photon flux and the corresponding dose impinging on the sample, depending on the absorption properties and the desired resolution. As an example, imaging a rather large micrometer-sized cell, like the widely used model system, Human-Embryo-Kidney (HEK) cells, requires $\approx 1\times 10^{14}$ of photons and a dose of ≈1.7 × 10^8^ Gy, for achieving a resolution of 50 nm using the zone plate imaging technique.^22,40^ Recent research in the hard X-ray regime revealed that the needed dose for the specimen could be decreased to a total of ≈10^4^ Gy (Ref. 41). Imaging the specimen, in the sub-micron regime, calls for lower photon numbers, but it must be always considered that this estimate holds for an illuminating beam covering only the area of the specimen. Generally, 10^11^ to 10^12^ photons per laser shot appear as the lower limit for meaningful imaging at a resolution better than an optical microscope. The above-mentioned minimum photon number for taking a single shot picture is only a basic requirement. A source must meet further requirements such as spatial and temporal coherence and stability. The only sources currently available for a single shot imaging in the water window are FELs. The combination of wiggling magnetic fields and relativistic electrons enables tunable sources with an extremely high brilliance up to $10^{34}\frac{\text{photons}}{s\cdot {\text{mrad}}^{2}\cdot {\text{mm}}^{2}\cdot 0.1\%\text{BW}}$ (FEL),^42^ outperforming any other X-ray source (Fig. 1). “Diffraction before destruction”-experiments, taking advantage of the femtosecond pulse duration of FELs, allowing to diffract light sufficiently before structural damage sets in, have been enabling advance in FEL imaging.^43–47^ The limited access to large-scale facilities is a driving factor for exploring the single shot imaging capability at the table-top scale. Laser produced plasma (LPP) XUV/SXR-sources in turn can generate almost sufficient photon flux for single shot imaging in the water window. However, their inherent low coherence prohibits the effective use for lensless imaging techniques, but they are ideally suited for lens-based imaging. For LPP, an intense laser is focused into a gas target^17,48,49^ or double gas target,^20^ attaining a flux of $1.51\times 10^{11}\frac{\text{photons}}{\text{pulse}}$^50^ in the water window. Using liquid nitrogen jets up to $1.1\times 10^{15}\frac{\text{photons}}{s\cdot \text{sr}\cdot \mu m^{2}\cdot \text{line}}$^16^ was demonstrated. Calculations from the study by O'Sullivan *et al.* in Ref. 51 for different other materials hold promise for new possibilities beyond the water window. A quick inspection reveals that the photon flux of these sources is a few orders of magnitude below the requirements for a single shot imaging micrometer-sized specimen.

**FIG. 2.**
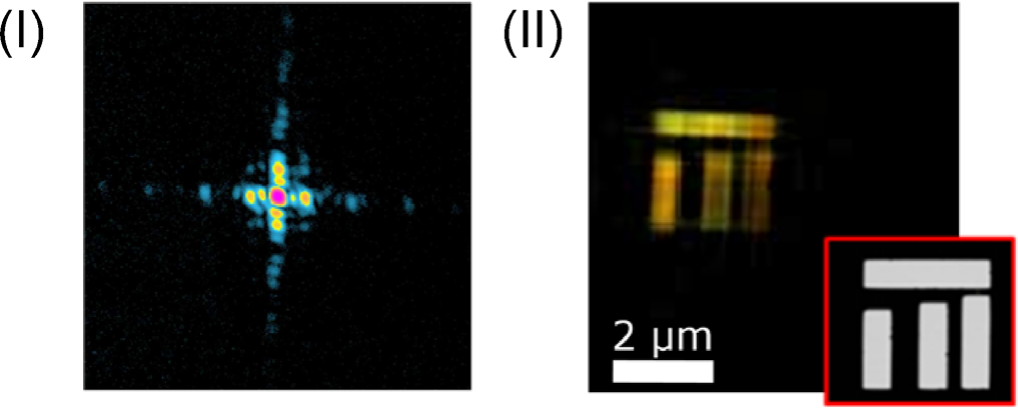
Capabilities of a SXRL at 32.8 nm by collisional ionization gating of krypton gas^24^ at the Laboratoire d'Optique Appliquée (Paris) with a single shot CDI setup of a binary object. (I) Diffractogram of the object [the SEM image is shown in the inset (II)]. (II) The reconstructed amplitude of the object from Ref. 90.

Lasers are the basis for realizing Soft-X-Ray-Lasers (SXRLs).^70^ There, a collisional excitation in the hot plasma can create inversion for core level transitions, resulting in laser-like emission of narrow-band XUV radiation with a flux sufficient for single shot imaging^71^ (Fig. 2). The rather low spatial coherence of the amplified spontaneous emission without the cavity significantly reduces the maximum object size for coherent microscopic imaging. However, seeding an SXRL with a high harmonic source was proven as a viable route for strongly enhanced coherent XUV radiation with femtosecond pulse duration and with a photon flux of $3\times 10^{11}\frac{\text{photons}}{\text{pulse}}$ at 32.8 nm.^24^ Such a high flux is sufficient for single shot imaging of objects with CDI. Although various routes for generating high harmonic radiation from ultrafast optical lasers are under investigation, to date, sources based on rare gas jets are dominating for imaging applications. The emission consists of multiple harmonics of the fundamental frequency, and the pulse duration can be shorter than the driving femtosecond laser pulses and as short as few tens of attoseconds.^23,72,73^ Additionally, the coherence properties of the driving laser beam are transferred to the harmonics. To optimize the conversion efficiency of HHG, various generation regimes for gas-phase HHG were realized, ranging from simple gas nozzles,^74^ semi-infinite gas cells^75^ to gas filled hollow core fibers.^76^ Each of them has advantages allowing to improve either the spatial properties or the selection of certain spectral components in the emitted radiation. Taking also the complexity and the reliability of the setup into account, the gas nozzle approach is the preferred method in many experiments aiming for generating ultrashort XUV/SXR-pulses for time-resolved spectroscopy and imaging. Using few cycle laser pulses and a gas nozzle allowed also for the first time HHG reaching the water window in 1997.^77^ More recently, it has been shown that shifting the driving laser wavelength into the mid-infrared, the short wavelength cut-off frequency of HHG is further pushed into the X-ray regime.^78–81^ Despite many improvements, a sufficient photon flux for single shot imaging could to date only be achieved at a wavelength below 40 nm. For this rather lower photon energy, first promising results have been obtained by Rupp *et al.*, demonstrating single shot imaging of helium nanodroplets^82^ and, Schwenke *et al.*, of a binary object with holography.^83^ Additionally, as recently demonstrated, the spatial coherence length of HHG radiation is inversely proportional to the harmonic order.^84^ For lensless imaging experiments, only the coherent fraction of the incoming XUV/SXR-radiation is useful, which will be significantly reduced at shorter wavelengths. Scaling HHG sources towards the X-ray regime is impeded by the significantly lower conversion efficiency^85^ and reduced spatial coherence at higher harmonic orders. Hence, meaningful routes to reach a sufficient photon flux are either higher energy driver pulses for single shot experiments or high average power driving sources^59^ for imaging applications permitting long exposure times. The currently commissioned Extreme Light Infrastructure beamline Attosecond Light Pulse Source (ELI-ALPS) is the most likely facility to reach sufficient parameters in the near future. With a flux high enough for single shot imaging,^86^ the facility offers beamlines with high repetition rates and stable attosecond XUV/SXR-pulses^87–89^ for spectroscopic and imaging applications. Although the first focus of ELI will not be water window single shot imaging, the facility and ELI framework hold great potential for such developments.

## IV. APPLICATION DEVELOPMENT

**FIG. 3.**
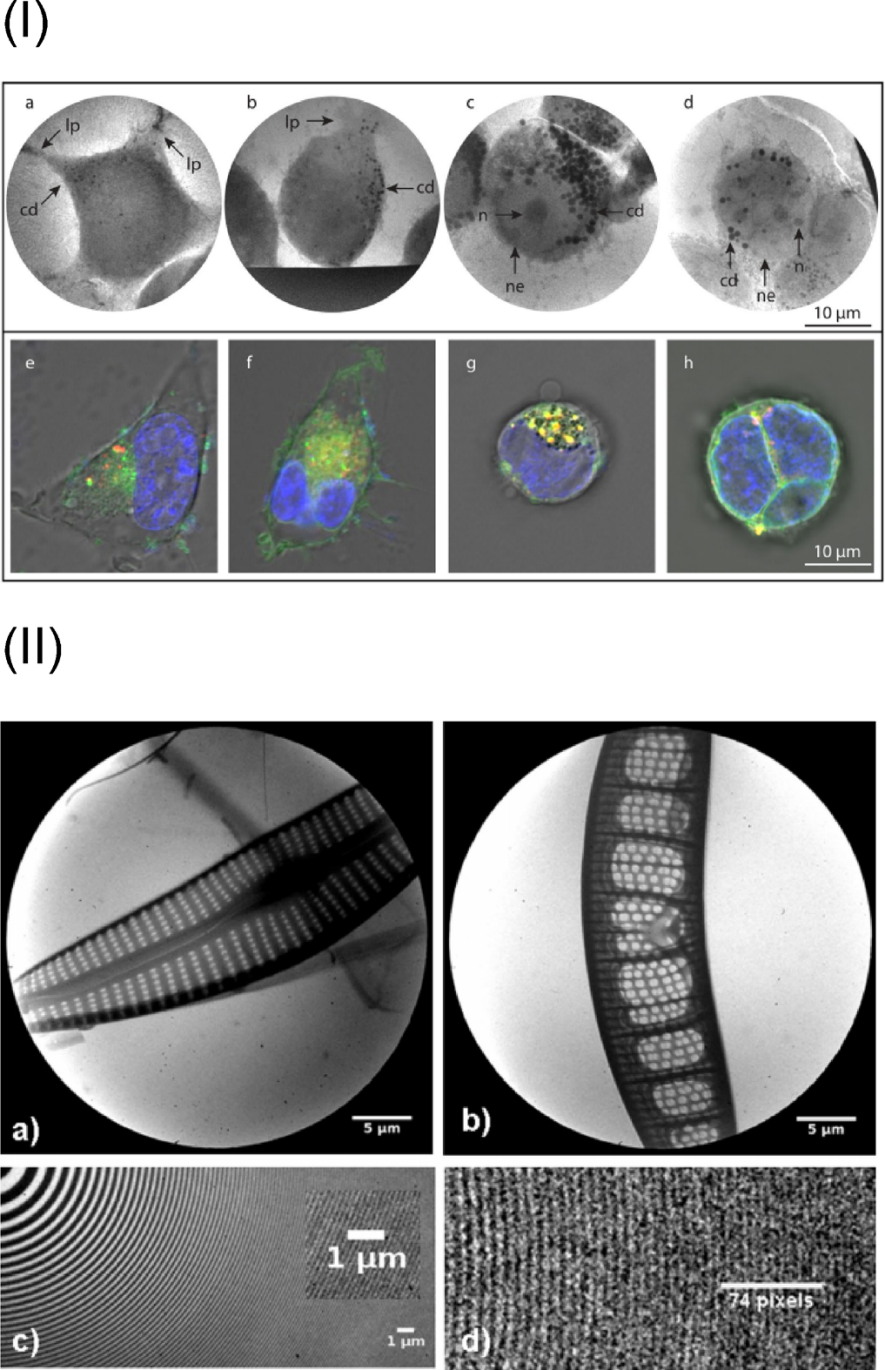
Examples of water window microscopy with a biological specimen. (I) Human-embryo-kidney cells at progression stages of starvation. Upper row [(a)–(d)]: Development of carbon-dense vesicles and rounding of the cell shape with ongoing time with a zone-plate cryo-X-ray-microscope. Lower row [(e)–(h)]: comparison to an optical confocal microscopy image with the same timeline of starvation. Reprinted with permission from Fogelqvist *et al.*, Sci. Rep. **7**, 13433 (2017). Copyright 2017 Authors, licensed under a Creative Commons Attribution 4.0 License. (II) Microscopy images of a diatom (upper row) with an acquisition time of 2 min and an image of the zone-plate in 3 min exposure time (lower row). Images were recorded with a lens-based microscope with a liquid-nitrogen plasma as the source in the water window. Reprinted with permission from Legall *et al.*, Opt. Express **20**, 18362 (2012). Copyright 2012 OSA.

The water window offers the possibility to gain insights into living materials. Fortunately, intense X-ray radiation and vacuum condition requirements do not infringe imaging a living specimen as shown in experiments at a FEL.^44,91^ In this work, the authors revealed the inner structure of biological samples with diffraction imaging in the before-destruction scheme. In such experiments, the biological samples are inserted via an injection device^92^ or fixed on a substrate by freezing the cells in their current state.^93^ The random orientation of the subsequently injected specimen allows for a three-dimensional reconstruction,^35,47^ by recording a large number of images to get a sufficient statistical distribution for the evaluation.^92^ The redundancy in the diffraction data allows assigning orientations for tomography via post-processing. For imaging a micron-sized specimen with table-top instrumentation (Fig. 3), the most appropriate approach is based on conventional zone-plate microscopy with discharge sources.^16,19,21,49,94^

**FIG. 4.**
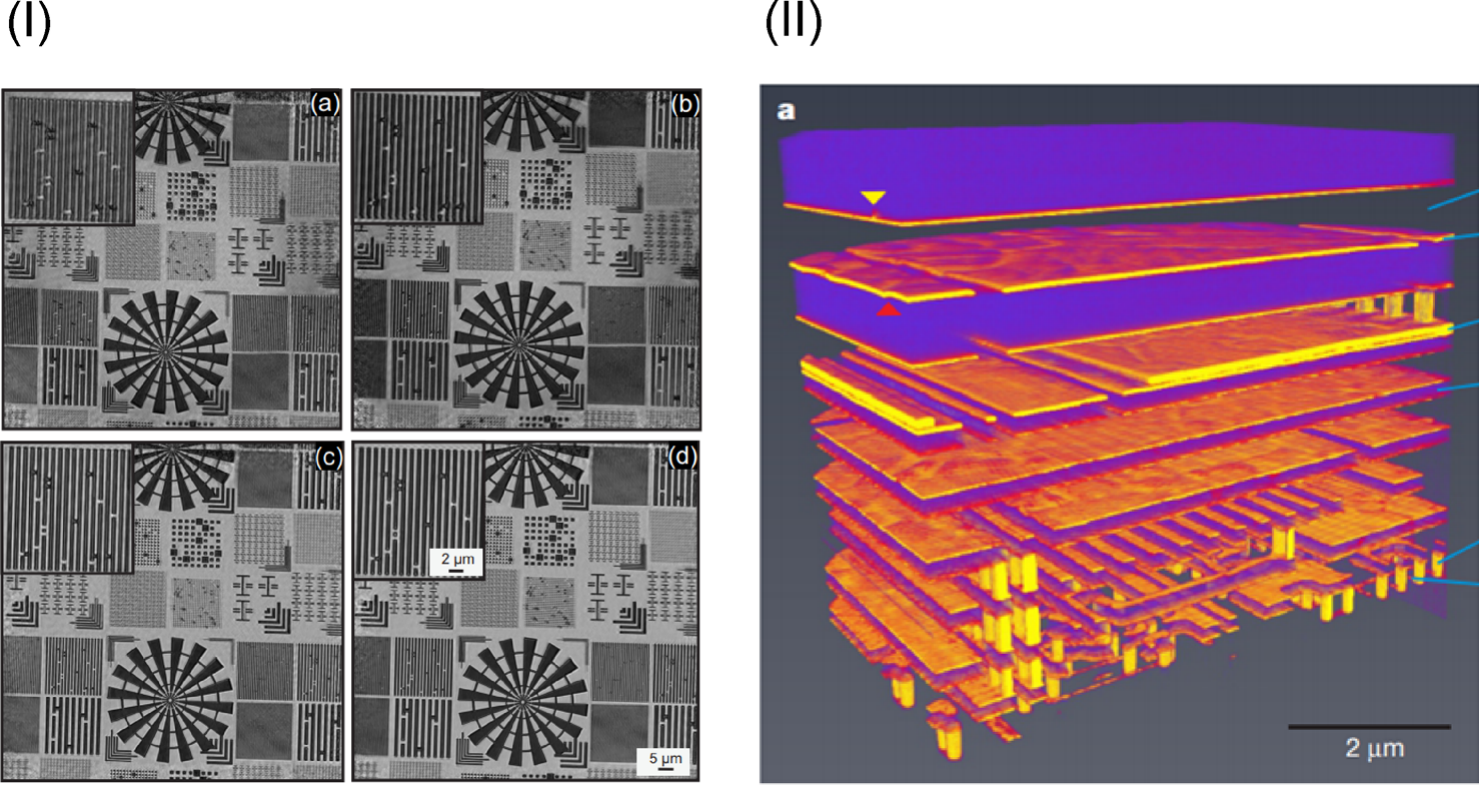
Example applications for the use of lensless imaging methods in the XUV/SXR-regime. (I) Reflection ptychography at an energy of 92 eV from a synchrotron with a different number of probes and modes of the beam. (a) 1 mode, 1 probe (b) 1 probe, 3 modes (c) 40 probes, 1 mode, and (d) 40 probes, 3 modes. Reprinted with permission from Helfenstein *et al.*, Opt. Express **26**, 12242 (2018). Copyright 2018 OSA. (II) Ptychographic 3D-X-ray-tomography of the application of a specific integrated circuit of a hybrid silicon pixel detector at an energy of 6.2 keV at a synchrotron. Reprinted with permission from Holler *et al.*, Nature **543**, 402–406 (2017). Copyright 2017 Authors, licensed under a Creative Commons Attribution 4.0 License.

It might take many more years until table-top sources are scaled to provide sufficient photon flux for imaging in the water window and even longer to reach single shot capability. These developments could be significantly accelerated according to needs of the semiconductor industry which is highly interested in table-top imaging se-ups for mask and defect inspection. High resolution XUV/SXR-imaging can provide unique capabilities for this since the short wavelength radiation has a large penetration depth and thus allows inspection of buried layers and structures, rendering the related methods superior to electron imaging approaches. Governed by Moore's law,^95^ the computer processor nowadays exhibits feature sizes well below 22 nm, prohibiting optical inspection methods in the visible region. In the production cycle, defects limit the effectivity of the processors and increase the cost for the manufacturer. The first 3D reconstruction of a processor structure performed by Holler *et al.*^37^ at a synchrotron showcases the extraordinary capabilities of XUV/SXR-imaging offers for the industry [Fig. 4 (II)].^96^ Further notable experiments at synchrotrons include the inspection of complete wafers with ptychography in the reflection mode [Fig. 4 (I)].^97^ For applications within the production sites, however, more compact sources are required, ideally implemented into lithography machines. First experiments with table-top sources show that defect inspections of masks combining CDI with HHG are possible.^34^ Zone-plate imaging with plasma discharge sources also enabled the investigation of lithography masks.^98–101^ Another approach for quality inspection uses a Shack-Hartmann-sensor to investigate a multilayer mirror.^102^ For applications in the semiconductor industry, sufficiently strong and compact table-top sources exploiting the silicon window around 100 eV photon energy are expected to become a reality soon, while in contrast to biological-related imaging, the accumulation of photons over several laser shots is not an intrinsic restriction. This leads to developments of sources that target high average flux rather than single shot capability.^63^ In one of the first successfully completed time-resolved experiments, the heat transport over nanoscale interfaces has been observed in a pump-probe imaging experiment.^103^ Observation of the magnetic domains of materials in the XUV/SXR-regime by FTH was first reported by Eisebitt *et al.* at a synchrotron.^25^ Since then, the development of circular polarized HHG offered new possibilities to investigate this phenomenon inside the lab.^104^ Traditionally, imaging experiments are performed in transmission geometry because it allows a simpler reconstruction procedure for CDI and ptychography.^105^ However, the transition from transmission experiments to reflection setups offers new possibilities for the direct measurement of changes and formations of the material, as it is well known for Laser Induced Periodic Surface Structures (LIPSS).^106^ LIPSS arise during the process of laser ablation or laser cutting, and the final structures has been extensively characterized with an atomic force or an electron microscope. However, their origin is still heavily debated in literature,^107–111^ calling for real-time observation to understand the formation dynamics of the characteristic ripples. The appearance of low and high spatial frequency LIPPS,^111^ with a grating period longer and shorter than the incident wavelength, respectively, can for instance be imaged with XUV/SXR pulses, providing a sufficient spatial and temporal resolution. Affected by the thickness of the object, a study in reflection geometry could stipulate progress in this field.

Increasing the brilliance of the sources is not the only possible route towards single shot recordings. Another promising approach is the structured illumination, which is the basis of many novel high-resolution microscopy approaches in the visible region such as STED. In contrast to the illumination with beams with a flat wavefront, singular light beams, which are solutions of the Helmholtz and Maxwell equations, offer new possibilities for improving the resolution^112^ in microscopy. The screw-like phase distribution, which is wrapped in a modulo 2*π* along the azimuthal coordinate, leads to a singularity in the center of the beam, i.e., the intensity vanishes. Such beams are called optical vortex beams and can be easily generated in the visible region by inserting into the beam path a spiral phase plate,^113^ a holographic phase imprint,^114^ or a spatial light modulator. Using such beams in an optical STED microscope, the resolution can be well below the Abbe limit. To exploit this approach in the XUV in a first proof-of-principle experiment, the transfer of optical vortices into the short wavelength range has been demonstrated,^115,116^ and generating beams with non-trivial wavefronts has sparked much interest recently.^117–119^ Attempts to use them for high resolution imaging in XUV/SXR are under way. Using the full spectrum of polychromatic XUV/SXR-sources is another possibility for increasing the photon flux on the sample. Using all harmonics of a HHG source limits the spatial resolution, due to the short coherence length, but increases the flux of the radiation. Several experiments with polychromatic light in a CDI or FTH scheme were successfully employed on binary objects.^120–126^ Limiting for this kind of imaging is the varying coherence of a group of harmonics.^84^ The superposition of the diffraction signals from several wavelengths in a single pixel of the detector must be carefully analyzed which is considered for reconstruction.

In this perspective, we have reviewed the tremendous progress in XUV/SXR-microscopy relying on imaging optics or lensless approaches using short wavelength light provided either from synchrotrons or laboratory sources. Many intriguing applications in physics, material science, biology, and medicine are underway. It will be very beneficial if the microscopic images can be recorded with a temporal resolution ranging from the second to attosecond range. Ideally, the image should be taken in a very short time to avoid blurring of the image due to faster than the exposure time processes and temporally accumulated radiation damage. Today, only FELs provide a necessary photon flux of roughly 10^14^ photons for single shot imaging in a few nanometer range. Further developments to advance laser driven XUV/SXR-sources based on HHG, SXRLs, and LPP emission hold great promise to come closer to the single shot regime in relevant wavelength ranges. These efforts include upgrading the existing driving lasers to deliver more intense pulses at higher repetition rates and, on the other hand, optimizing the conversion process to produce XUV/SXR radiation at shorter wavelengths with higher efficiency and improved spatial and temporal properties. A dominant current direction of research pursued in many groups with the first systems being commercially available involves the development of high-energy optical-parametric chirped pulse amplification laser systems capable of generating multi-millijoule pulses in the near infrared, which provides potential for generating intense soft X-rays using HHG. These developments hold great promise to enable table-top microscopes meeting the requirements for single shot imaging of biological relevant specimens and for time-resolved imaging of the spatial modifications of inorganic samples.
